# Investigating the non-specific effects of BCG vaccination on the innate immune system in Ugandan neonates: study protocol for a randomised controlled trial

**DOI:** 10.1186/s13063-015-0682-5

**Published:** 2015-04-11

**Authors:** Sarah Prentice, Emily L Webb, Hazel M Dockrell, Pontiano Kaleebu, Alison M Elliott, Stephen Cose

**Affiliations:** Wellcome Trust - Bloomsbury Centre for Global Health Research, London School of Hygiene and Tropical Medicine, Keppel Street, London, WC1E 7HT UK; Clinical Research Department, London School of Hygiene and Tropical Medicine, Keppel Street, London, WC1E 7HT UK; Department of Infectious Disease Epidemiology, London School of Hygiene and Tropical Medicine, Keppel Street, London, WC1E 7HT UK; Department of Infection and Immunology, London School of Hygiene and Tropical Medicine, Keppel Street, London, WC1E 7HT UK; MRC/Uganda Virus Research Institute on AIDS, Plot 51-59, Nakiwogo Road, PO Box 49, Entebbe, Uganda

**Keywords:** Bacillus Calmette-Guérin, Heterologous effects, Innate immunity, Neonate, Invasive infectious disease

## Abstract

**Background:**

The potential for Bacillus Calmette-Guérin (BCG) vaccination to protect infants against non-mycobacterial disease has been suggested by a randomised controlled trial conducted in low birth-weight infants in West Africa. Trials to confirm these findings in healthy term infants, and in a non-West African setting, have not yet been carried out. In addition, a biological mechanism to explain such heterologous effects of BCG in the neonatal period has not been confirmed. This trial aims to address these issues by evaluating whether BCG non-specifically enhances the innate immune system in term Ugandan neonates, leading to increased protection from a variety of infectious diseases.

**Methods:**

This trial will be an investigator-blinded, randomised controlled trial of 560 Ugandan neonates, comparing those receiving BCG at birth with those receiving BCG at 6 weeks of age. This design allows comparison of outcomes between BCG-vaccinated and -naïve infants until 6 weeks of age, and between early and delayed BCG-vaccinated infants from 6 weeks of age onwards. The primary outcomes of the study will be a panel of innate immune parameters. Secondary outcomes will include clinical illness measures.

**Discussion:**

Investigation of the possible broadly protective effects of neonatal BCG immunisation, and the optimal vaccination timing to produce these effects, could have profound implications for public healthcare policy. Evidence of protection against heterologous pathogens would underscore the importance of prioritising BCG administration in a timely manner for all infants, provide advocacy against the termination of BCG’s use and support novel anti-tuberculous vaccine strategies that would safeguard such beneficial effects.

**Trial registration:**

ISRCTN59683017: registration date: 15 January 2014

**Electronic supplementary material:**

The online version of this article (doi:10.1186/s13063-015-0682-5) contains supplementary material, which is available to authorized users.

## Background

### Background and rationale

Bacillus Calmette-Guérin (BCG) immunisation, the only currently available tuberculosis (TB) vaccine, is one of the most frequently administered immunisations worldwide with more than 100 million children receiving it per year [1]. Although it provides protection against severe forms of TB in children, it has variable efficacy against adult pulmonary disease, with protection generally poor in high-risk areas such as sub-Saharan Africa and Asia [2]. There are currently concerted efforts in the scientific community to improve anti-TB protection either by enhancing existing BCG immunisation strategies or by developing an alternative vaccine [3].

However, it has been suggested that BCG may protect infants against a variety of non-mycobacterial pathogens and thus have beneficial effects beyond protection against TB [4]. The evidence for such a ‘non-specific’ effect of BCG is currently in equipoise. It is, therefore, important and pressing to interrogate this possibility further so that any new vaccine or BCG schedule may be evaluated in terms of overall benefit to recipient, rather than in terms of TB-specific protection alone.

The possibility that BCG may have non-specific beneficial effects on diseases other than TB has been a controversial and highly-debated subject. Observations that BCG may have a greater impact on mortality than can be explained by protection against TB were first made following its introduction more than 80 years ago. Studies including more than 46,500 infants, carried out in the 1940s and 1950s in the USA and UK, showed on average a 25% (95% CI 6 to 41%) reduction in all cause mortality in children receiving BCG compared to those not receiving it [5-9]. This reduction was noted at the time to be larger than could be attributed to the expected reduction in rates of TB. However, as many of these studies were not strictly randomised or controlled, and this was a period of major public health improvements, the results were assumed to result from confounding effects. Similar arguments have been used to dismiss a number of observational studies carried out more recently, in Guinea-Bissau, which appear to show that infants who receive BCG at birth have lower all-cause morbidity and mortality than infants who do not [10-17].

Good quality, randomised controlled trials evaluating the possibility of non-specific effects of BCG are extremely limited. Only one trial has been conducted to specifically evaluate non-tuberculous mortality as a result of altered BCG vaccination schedule [18]. In this trial of low birth-weight infants in Guinea-Bissau, subjects randomised to receive BCG at birth had a 45% lower mortality rate (MRR 0.55 (0.34 to 0.89)) in the first 2 months of life than infants who had BCG immunisation delayed to, on average, 6 weeks of age. The reduction in deaths was due to protection from all-cause febrile illness, respiratory tract infections and diarrhoea, but not against TB (verbal autopsy data). However, although this study is the only trial designed primarily to investigate the impact of BCG on all-cause mortality, nine other randomised controlled trials have been conducted that delayed BCG vaccination past the neonatal period in high mortality areas [19-27]. None of these studies reported significant differences in mortality, either during the period when one intervention group had received BCG and the other group had not, or subsequently. Also, in contrast to the Guinea-Bissau trial, three large cohort studies appear to show that infants who receive BCG vaccination at the same time as Diphtheria Tetanus Pertussis (DTP) vaccination (at 6 weeks of age) have reduced longer-term all-cause mortality than those that have received BCG at birth [28]. Thus, it is currently unclear whether BCG has non-specific beneficial protective effects against diseases other than TB, and if so, what timing of administration would be optimal to induce these effects.

The possibility that BCG may have effects against non-tuberculous disease has also had limited acceptance in the scientific and public health communities due to the lack of a confirmed biological mechanism. Investigations into the hypothesis that BCG immunisation might skew the adaptive immune response from the T-helper type 2 (Th2) dominant environment of early neonatal life, toward a more protective T-helper type 1 (Th1) environment, have been inconclusive [29-32]. The evidence from the Guinea-Bissau randomised controlled trial, however, shows that any putative immunological mechanism would need to be: 1) effective at birth despite the immature neonatal immune system, 2) rapidly inducible (most protection at < 1 week post-immunisation) and 3) active against a range of pathogens. These features would suggest that BCG mediates its non-specific effects by stimulating the innate immune system. This is the hypothesis that we aim to interrogate during this study.

We plan to investigate three different aspects of the innate immune system. Firstly, we will investigate whether non-specific pro-inflammatory cytokine production is enhanced in infants who have received BCG by using *in vitro* stimulation with non-mycobacterial stimulants. Few studies exist investigating alterations in cytokine production to heterologous stimulants following neonatal BCG immunisation [33-35]. The few that have been reported have focused on adaptive cytokines, using a 6-day *in vitro* stimulation protocol, which is sub-optimal for the quantification of innate cytokine production. No studies exist where samples have been collected prior to 5 months of age, thus early non-specific effects of BCG will have been missed. Lastly, *in vitro* stimulants used in previous studies have been antigens (for example, lipopolysaccharide or tetanus toxoid) and not whole organisms, potentially excluding the effect of other important pattern recognition receptor pathways. As part of this proposed study we aim to address these issues by focusing on the impact of BCG on innate cytokine production, conducting overnight stimulation using non-mycobacterial whole organism stimulants, and by using blood samples taken before 10 weeks of age.

Secondly, we will investigate whether BCG might mediate any non-specific beneficial effects by inducing a plasma hypoferraemia. Iron supply is critical for the virulence of most pathogens [36], with plasma hypoferraemia profoundly inhibiting the growth of bacteria [37,38], viruses [39], protozoa [40-43] and fungi [44,45]. As part of the innate acute-phase response, plasma hypoferraemia is induced by IL-6-driven release of hepcidin. Guinea pig models reveal that BCG also induces a rapid bacteriostatic hypoferraemia [46], although involvement of the IL-6/hepcidin pathway has never been studied. To our knowledge, no studies exist investigating the influence of BCG immunisation on the human iron-inflammatory pathway. As part of this study we will investigate whether BCG immunisation in neonates induces alterations to the inflammatory iron axis, as a potential effector mechanism for heterologous protection.

Lastly we will investigate whether BCG induces epigenetic modification at the promoter region of pro-inflammatory cytokines in monocytes, thereby providing a mechanism for ‘training’ the innate immune system to respond in a persistently amplified manner to challenge by non-mycobacterial pathogens. BCG immunisation of naïve adults has been shown to produce trimethylation of histone-3 lysine 4 (H3K4) at the promoter region of TNF-α, IL-1β and IFN-γ in monocytes [47]. This led to enhanced cytokine production following *in vitro* stimulation with the heterologous pathogens *Staphylococcus aureus* (*S. aureus*)*, Streptococcus pneumonia* (*S. pneumoniae*) and *Candida albicans* (*C. albicans*)*,* which persisted to at least 3 months post-immunisation. We will investigate whether BCG immunisation produces similar epigenetic modification of monocytes in neonates.

Thus, we have designed a randomised controlled trial, comparing BCG administration at birth with administration at 6 weeks of age in healthy Ugandan neonates. We will use this to interrogate the impact of BCG vaccination on the innate immune response, as well on all-cause clinical illness outcomes. We believe this study will add significantly to the current debate regarding the non-specific effects of BCG vaccination as it aims to confirm a biological mechanism to explain such effects. Also, by being conducted in healthy neonates, in a geographical location distant from previous studies and by an independent research group, it will help to understand the global applicability of any non-specific effects.

### Aims and objectives

The aims of our study are as follow:

1. To determine whether BCG immunisation at birth alters the innate immune response to heterologous pathogens in the short term (within 1 week)
2. To determine whether BCG immunisation at birth alters the innate immune response to heterologous pathogens in the longer term (at 6 weeks)
3. To determine whether BCG immunisation given at age 6 weeks has similar short- and longer-term effects on the innate immune response to heterologous pathogens compared to BCG immunisation at birth
4. To obtain data upon the effect of BCG on neonatal susceptibility to invasive infections in Ugandan infants

Aims 1, 2 and 3 will be addressed using sub-studies to interrogate 3 different elements of the innate immune system. The individual objectives for these studies are shown in Table 1. Clinical outcome measures from all 3 sub-studies will be combined to address Aim 4.

**Table 1:** **Objectives for immunological sub-studies**

**Sub-study**	**Primary objectives**	**Secondary objectives**
Cytokine sub-study	Cross-sectional comparison of IL-1β, IL-6, TNF-α and IFN-γ cytokine levels following overnight *in-vitro* stimulation with *S. aureus, S. pneumoniae, E. coli, C. albicans* and Poly I:C/CpG between the two intervention groups:	Longitudinal analysis of within-infant changes in innate cytokine production following *in-vitro* stimulation with *S. aureus, S. pneumoniae, E. coli, C. albicans* and Poly I:C/CpG.
	1. Shortly after birth intervention (BCG vaccination/no vaccination): Aim 1	
	2. Six weeks post-birth intervention (immediately prior to first dose of primary vaccination): Aim 2	
	3. Shortly after 6-week intervention (BCG vaccination/no vaccination): Aim 3	
	4. Three weeks post-6-week intervention (immediately prior to second dose of primary vaccinations): Aim 3	
Iron sub-study	Cross-sectional comparison of transferrin saturation and hepcidin levels between the two intervention groups:	Cross-sectional comparison of serum iron, total iron binding capacity, ferritin, transferrin, haemoglobin and red cell parameters at the above time-points.
	1. Shortly after birth intervention (BCG vaccination/no vaccination): Aim 1	Longitudinal analysis of within-infant changes to iron status following *in-vivo* non- specific stimulation (provided by primary vaccinations)
	2. Six weeks post-birth intervention (shortly after first dose of primary vaccination): Aim 2	
	3. Shortly after 6-week intervention (BCG vaccination/no vaccination): Aim 3	
	4. Three weeks post-6-weeks intervention (shortly after second dose of primary vaccination): Aim 3	
Epigenetic sub-study	Cross-sectional comparison of monocyte histone-3 lysine 4 trimethylation (H3K4me3) at the promoter region of pro-inflammatory cytokines between the 2 intervention groups:	Longitudinal analysis of within-infant changes in monocyte epigenetic modification.
	1. Shortly after birth intervention (BCG vaccination/no vaccination): Aim 1	
	2. Six weeks post-birth intervention (immediately prior to first dose of primary vaccination): Aim 2	

### Study design

This study is an investigator-blinded randomised controlled trial of BCG vaccination given at birth versus BCG vaccination given at 6 weeks of age. Cord blood and two venous blood samples will be collected from participants to allow comparison of innate immune system parameters. All participants will be clinically followed-up until 10 completed weeks of age, to allow comparison of illness outcomes. This study design will allow comparison of outcomes between BCG-naïve and -vaccinated infants up to 6 weeks of age, and early with delayed BCG-vaccinated infants from 6 to 10 weeks of age, helping to identify whether there is a critical period for BCG-induced non-specific effects. The time-point of 6 weeks for the delayed BCG group has been chosen as it is the longest delay possible prior to the potential confounding influence of primary immunisations.

## Methods

### Setting and participants

Infants will be recruited on the day of birth from the maternity ward of Entebbe Grade B hospital, a government hospital located in Wakiso District in central Uganda. The region is populated mainly by semi-urban, rural and fishing communities. Neonatal mortality rates in Uganda remain high at 28/1,000 live births, with a large proportion attributable to invasive infectious diseases.

### Eligibility criteria

The inclusion criteria for this study are:

1. Infant of a gestational age and birth weight sufficient to allow discharge directly home from hospital without requirement for supplemental oxygen or feeding
2. Delivery sufficiently uncomplicated to allow discharge directly from hospital without inpatient management
3. HIV-negative mother (based on antenatal records)
4. Residence within the study catchment area
5. Consenting mother

No specific weight or gestational age limit has been set for this study. Clinical responses to early BCG are suggested to have the greatest effect in infants of the lowest birth weight [18]; thus, it is important to include these infants in data collection. No increased rate of detrimental side-effects or reduction of immunological efficacy has been shown with BCG immunisation of premature infants [48]. Written informed consent will be obtained from the mothers of all infants prior to their enrolment in the study.

Neonates will be excluded from the study if:

1. Cord blood is not obtained
2. They have major congenital malformations
3. The infant is clinically unwell, as judged by a midwife
4. Known maternal TB or active TB within the family (based on direct questioning of mother during recruitment)
5. Maternal or family member positive for any of the following TB screening symptoms:
  Cough > 2 weeks
  Recent haemoptysis
  >3 kg weight loss in past month
  Recurrent fevers/chills or night sweats for the past 3 days or more

### Intervention and randomisation

All infants will receive 0.05 ml of BCG-Statens Serum Institute (SSI, Copenhagen, Denmark) (Danish Strain 1331) intra-dermally into the right deltoid. This will be given either at birth (Early intervention arm) or at 6 weeks of age (Delayed intervention arm).

Intervention and blood sampling time-point allocation will be determined by block randomisation, stratified by sex. This will be carried out by an independent statistician, prior to the trial commencement, using Stata (StataCorp, College Station, TX, USA) to generate the allocation sequence. Allocations will be concealed within sequentially numbered, sealed opaque envelopes, prepared by two research assistants who are independent of the trial. Upon delivery of an eligible infant, assignment of allocation will be carried out by midwives who will select the next sequential envelope according to the infant’s gender.

### Blinding

This study will be single blind. Mothers will not be blinded to intervention allocation due to lack of feasibility (BCG produces a visible reaction) and to reduce confusion if a child who is lost to follow-up presents to a community immunisation clinic.

Staff involved in administering BCG immunisation to the participants, either at birth or at 6 weeks of age, will not be involved in clinical follow-up or assessment of outcomes.

Investigators performing clinical assessment of children will be blinded to intervention allocation by means of a plaster placed over the area corresponding to BCG vaccination site. This will be placed by a nurse not involved in clinical assessment, prior to physician assessment. If a child is presenting due to concerns about the immunisation site it will be left uncovered and the un-blinding documented. Illness events arising from concerns or complications directly related to the BCG immunisation will not be included in the analysis of illness events, but will be presented separately.

Immunological investigations will be conducted on blood samples identified only by study number. The intervention allocation code will only be broken once laboratory analysis is complete and the data have been cleaned and locked.

### Study procedures

#### Overview

**Figure 1:**
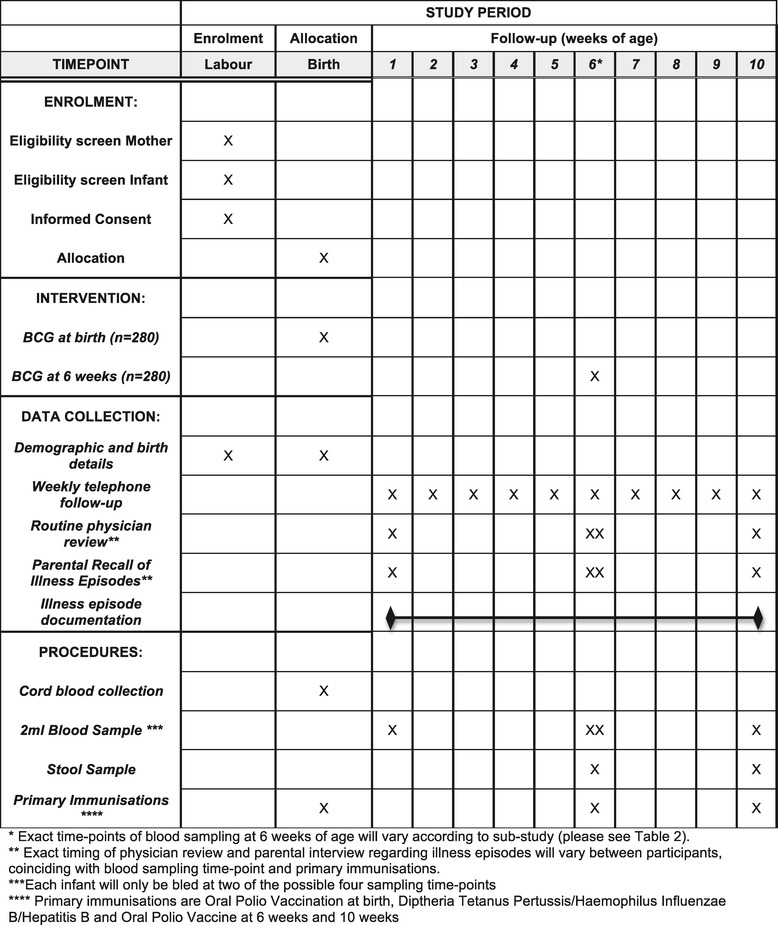
**Standard Protocol Items: Recommendations for Interventional Trials (SPIRIT) diagram of study procedures.**

Figure 1 shows the SPIRIT (Standard Protocol Items: Recommendations for Interventional Trials) diagram for the trial procedures. On presentation to labour ward, mothers in active labour will be screened for their eligibility and informed consent will be taken. Following delivery the infant will be assessed for eligibility and placental cord blood collected. Infants who are eligible for the study will be randomised as described above, to receive BCG vaccination either immediately or at 6 weeks of age. All infants will be followed-up until 10 completed weeks of age. During this time 2 × 2 ml venous blood samples and 2 stool samples will be collected and all routine immunisations will be given (Oral Polio Vaccination (OPV) at birth and primary immunisations at 6 and 10 weeks of age). Clinical follow-up of the infants will be carried out by weekly telephone interviews to check the well-being of participants, and physician review and anthropometry at each routine clinic visit for blood samples/routine immunisations (on average four visits per participant). Unwell participants presenting to the study clinic or Entebbe Grade B hospital will also be reviewed and managed by the study team, free of charge. Study follow-up is complete once the child has completed 10 weeks of age.

### Consent

Sensitisation of parents to the study will occur during antenatal classes via posters, group discussions and during individual midwife-led consultations. Mothers will then be approached for consent by trained midwives when presenting in active labour to Entebbe Grade B hospital. The study will be explained in detail verbally and the information sheet provided (or read to illiterate mothers). Information sheets will be available in English and Luganda. Consent will also be taken to allow for storage of excess samples and use of data in future research studies. Although consent during labour is not optimal, it is necessary to enable cord blood collection. However, consent will be verbally reconfirmed with mothers following delivery prior to any intervention. This method of consent and recruitment has been piloted in the same hospital and shown to be an appropriate and successful method.

### Data collected

Demographic details, anthropometric measurements and socio-economic indices will be collected at enrolment including gender, gestational age, birth weight, occipito-frontal circumference and length, maternal age and parity, parental ethnicity, parental educational level attained, accommodation type and assets. Global Positioning System (GPS) co-ordinates of the participant’s home address will also be collected to aid follow-up.

During routine clinic visits anthropometric and vital sign measurements will be collected. All mothers will be interviewed about illness episodes in the participant since they were last seen in clinic and any current concerns. Physical examination findings will be documented.

A standardised illness episode case report form will be completed whenever a child presents unwell to the research clinic or paediatric ward at Entebbe Grade B hospital. This will include anthropometric and vital sign measurement, symptoms and signs, investigation results, final diagnosis and outcome.

All participants will be interviewed by telephone on a weekly basis by a fieldworker using a standardised case report form to ensure the health of the infant. Any infants for whom there are concerns will be reviewed in clinic. This intensive follow-up will enhance identification of clinical illness episodes, which are secondary outcomes for the study. More importantly, however, it will allow early identification and management of any cases of perinatal TB, particularly in the delayed intervention group. Any suspected or confirmed cases of TB occurring during the study will be reported to the ethics committees and Data Safety Monitoring Board (DSMB), who will decide whether the study needs to be stopped early for safety.

Direct electronic data entry will occur for all case report forms. This will be verified and optimized by co-documentation with paper case report forms at the beginning of the study. Data will be maintained in encrypted, password protected forms, to maintain confidentiality.

### Blood samples collected

All participants in the study will have 10 ml placental cord blood collected at birth; divided into 5 ml of heparinised and 5 ml of ethylenediaminetetraacetic acid (EDTA)-anticoagulated blood. They will then have 2-ml venous blood samples collected at 2 time-points between birth and their exit from the study at 10 completed weeks of age. Each sub-study has up to four possible time-points where blood samples are collected, but each infant will only be bled at two of these time-points (randomly allocated) to avoid undue stress for the baby and the mother. The time-points have been selected to enable interrogation of the changes to the innate immune system induced by BCG both acutely following vaccination and in the longer term. The timing of the blood samples in the iron sub-study differs slightly from those in the cytokine and epigenetic sub-studies (see Table 2). These differences are necessitated by the systemic nature of iron metabolism. As hepcidin is produced mainly in the liver this precludes analysis of iron metabolism following *in-vitro* non-specific stimulation. Thus, the iron sub-study will use routine primary immunisations as *in-vivo* non-specific stimuli and measure the resulting changes to iron parameters.

**Table 2:** **Blood sample time-points (T) according to immunological sub-study**

	**Blood sample T1 (first week of life)**	**Blood T2 (6 weeks of age)**	**Blood sample T3 (6 weeks of age)**	**Blood sample T4 (10 weeks of age)**
Cytokine sub-study	5 days after birth	Immediately before primary immunisations	5 days after primary immunisations	Immediately before primary immunisations
Iron sub-study	5 days after birth	1 day after primary immunisations	5 days after primary immunisations	1 day after primary immunisations
Epigenetic sub-study	5 days after birth	Immediately before primary immunisations		

### Stool samples

Stool samples will be collected at the 6-week and 10-week time-points and stored to allow for future analysis, funding permitting.

### Other samples collected

Whenever an unwell participant presents to the study team investigations and treatments will be conducted as directed by the attending clinician. Investigations will include cultures for accurate diagnosis of febrile illness. An extra 2-ml blood sample will be taken from any participant under-going phlebotomy provided that this will not compromise the child’s health or well-being. This will allow a sub-study to be conducted to compare primary immunological outcomes in unwell children according to BCG status.

### Laboratory procedures

#### Cytokine sub-study

Overnight whole blood stimulation with the non-specific stimulants *S. aureus, S. pneumoniae, E. coli, C. albicans* and polyinosinic:polycytidylic acid/C-phosphate-G (Poly I:C/CpG) will be carried out using fresh sodium-heparinised blood. Measurement of the pro-inflammatory cytokines IL-1β, IL-6, TNF-α and IFN-γ by ELISA (BD-OptEIA, Becton, Dickinson and Company, Oxford, UK) will then be conducted on the harvested supernatant following storage at −80°C. These stimulants have been chosen because they are the most common pathogens isolated from septic neonates in Uganda [49] and because they represent a range of pathogen types and toll-like receptor pathways.

#### Epigenetic sub-study

The levels of trimethylation of H3K4 at the promoter region of pro-inflammatory cytokines will be assessed using chromatin immunoprecipitation followed by qPCR. Peripheral blood mononuclear cell (PBMC) isolation for this work will occur by density-centrifugation over histopaque (Sigma-Aldrich, Dorset, UK).

#### Iron sub-study

Measures of iron status will be conducted on the plasma fraction of lithium-heparinised blood following storage at −80°C. Serum iron, Unbound Iron Binding Capacity (UIBC), Total Iron Binding Capacity (TIBC), Transferrin Saturation (TSAT) and ferritin will be measured using the automated Cobas Integra (Roche Diagnostics, Switzerland). The hormone hepcidin will be quantified using ELISA (Bachem-25, Bachem, Switzerland).

Red cell parameters will be measured from fresh EDTA whole blood using a Coulter A^**C.**^T 5 Diff CP haematology analyser (Beckman Coulter, Inc, CA, USA).

### Primary outcomes

#### Cytokine sub-study

- IL-1β, IL-6, IL-10, TNF-α and IFN-γ cytokine levels following *in-vitro* stimulation with *S. aureus, S. pneumoniae, E. coli, C. albicans* and Poly I:C/CPG*.*

#### Epigenetic sub-study

- H3K4 trimethylation at the region of pro-inflammatory cytokines in peripheral blood monocytes

#### Iron sub-study

- Hepcidin levels
- TSAT

Primary outcomes in each sub-study will be compared between the 2 intervention groups both acutely following BCG (up to 1 week after birth/6 weeks of age) and at time-points distant from vaccination (6 and 10 weeks of age).

### Secondary outcomes

- Physician-diagnosed infectious disease
- Parental-reported infectious disease
- Culture-positive infectious disease
- Mortality

The above clinical outcomes for the three sub-studies will be analysed together to increase power.

The iron sub-study will also have the following secondary outcomes:

- Serum iron
- TIBC
- Ferritin
- Transferrin
- Haemoglobin
- Red cell parameters

In a secondary analysis, longitudinal within-infant changes in primary outcomes will also be analysed for each sub-study.

### Sample size considerations

Each sub-study is powered for its own primary outcomes. The overall sample size is the summation of the participants required for each sub-study.

#### Cytokine sub-study: n = 240

Due to paucity of published data in this area, an approach based on standard deviation (SD) difference in average population cytokine levels has been used. Forty-eight subjects per intervention group (BCG immunisation at birth or at 6 weeks of age) will be needed at each time point to show a 0.66 SD difference in average population cytokine levels with 90% power and 5% significance. Sixty infants per intervention group per time point will be recruited to allow for attrition. As each recruited infant will be bled at 2 time-points, 240 infants will be recruited in total to allow for the 4 time-points.

#### Epigenetic sub-study: n = 80

The only previous study in this area (which was performed in adults) required 20 subjects per intervention arm [47]. We will recruit 40 subjects to each intervention arm to allow for attrition and also due to the requirement for a full 2-ml blood sample for epigenetic analysis, which is unlikely to be obtained for all subjects. Due to funding constraints, epigenetic analysis will be restricted to the first two sampling time-points, and each infant will be bled at both time-points, eighty subjects will be recruited in total.

#### Iron sub-study: n = 240

Sample size determination was performed using TSAT as it is the only primary outcome parameter currently of clinical relevance. Average neonatal TSAT in low-income settings is 55% [50]. Fifty infants in each group at each time point will be needed to show a 30% reduction in transferrin saturation (reduction to average TSAT levels in low income settings) with 90% power and 5% significance. Sixty subjects will be recruited to each intervention group at each time point to allow for attrition. As each recruited infant will be bled at 2 time-points, 240 infants will be recruited in total.

#### Overall sample size: n = 560

Combined analysis of clinical end-points from all three sub-studies will be conducted as secondary analysis. Based on data from a previous study in Entebbe [51] we expect 80% power to detect a ≥ 40% reduction in physician-diagnosed invasive infections with 5% significance. The effect of BCG is unlikely to be this pronounced, but this preliminary data combined with the primary immunological outcomes, should provide sufficient evidence to determine whether expanding the cohort would be valuable.

### Data management

#### Description of the data

This is a randomised controlled trial with datasets generated from clinical questionnaires and laboratory assays. A combination of direct electronic capture and paper forms will be used, linked by a unique participant identifier. Microsoft Access (Redmond, WA, USA) will be utilised to produce the study database. Data will be exported from Microsoft Access to Stata (StataCorp, College Station, TX, USA) for statistical analysis.

#### Quality assurance

A detailed data dictionary with range checks will be used to reduce data entry errors. Quality control checks will be run by the data clerk, on a weekly basis, who will highlight any queries to the principal investigator. Data will only be uploaded onto the master database once any queries highlighted by quality control checks have been resolved.

### Statistical analysis

Group characteristics will be compared using Pearson’s Chi-squared test for categorical variables and the *t*-test for continuous variables. Cross-sectional comparisons between intervention groups at each time-point will be carried-out using the *t*-test for differences between means. Non-normally distributed outcome data will be log-transformed before analysis; Mann–Whitney two-tailed test will be used for persistently skewed data. If potential confounders remain unbalanced between the groups despite randomisation: for instance season of birth, these will be adjusted for using multiple linear regression analysis. Paired/longitudinal analysis of within infant changes in parameters over time will be conducted using the paired student *t*-test or Wilcoxon matched-pairs test. Incidence rate of invasive infectious disease in the first 10 weeks of life will be compared by Poisson regression with a random effects model to allow for within-child clustering. Statistical significance will be assessed at the 2-sided 0.05 level but interpretation of results will not be solely reliant on *P*-values.

### Trial monitoring

This clinical trial will be conducted according to Good Clinical Practice standards. An internal study monitor will oversee the day-to-day running of the trial locally, with external oversight and monitoring co-ordinated by the London School of Hygiene and Tropical Medicine. This may include internal audit by the Clinical Trials Quality Assurance Manager and external audits by a third party. A Trial Steering Committee (TSC) and an independent DSMB have been set up for this study. The DSMB will look at a number of clinical outcome measures, documented in 'real time' during the study, to assess whether the study needs to be stopped early for safety.

Safety reporting for this trial will follow standard Uganda Virus Research Institute and London School of Hygiene and Tropical Medicine procedures. This includes notification of Serious Adverse Events (SAEs) to the local ethics committee within 24 hours, notification of Suspected Unexpected Serious Adverse Reactions (SUSARs) to the sponsor within 7 days if life-threatening or 15 days if non-life-threatening. The manufacturer of the BCG vaccine, Staten Serum Institute, will also be notified of any SAE/SUSAR.

### Ethics

As this trial will alter the timing of BCG from the current Ugandan guidelines (BCG at birth) in half of the study infants, a thorough risk-benefit analysis of a 6-week delay in vaccination has been conducted. In summary, we feel that the risks of delay are minimal for the following reasons:

- Neonatal TB is rare and the chances of infants in the delayed BCG arm becoming infected during a 6-week delay period are extremely small. At least 7 previous studies have been conducted in areas of high TB prevalence that randomised infants to delayed BCG vaccination past 6 weeks of age [19-24]. None of these studies showed an increase in TB incidence in the delayed vaccination group either in the period prior to vaccination or during follow-up (cumulative n for delayed BCG vaccination = 849, median follow-up time 1 year).
- A recent study using an Entebbe based birth-cohort showed a prevalence of latent TB infection of 9.7% in children under 5 years old [52]. This suggests that in our population, a 6-week delay in BCG administration risks 0.63 infants becoming infected with latent TB. However, the strongest risk factor for latent TB acquisition in Entebbe is a known contact with a TB case (odds ratio (OR) 2.62 (1.29 to 5.30), unpublished data). Thus, the exclusion of infants at risk of TB from mother or a household contact will reduce this risk to negligible. Active weekly follow-up of infants will occur to ensure they remain healthy and the trial will be stopped early if cases of TB are found to be higher in the delayed BCG arm.

There is also evidence that delay in BCG vaccination from birth to 6 weeks may be beneficial for participants because:

- The optimal timing of BCG vaccination for immunity against TB is not known. There is some evidence that delaying BCG past the neonatal period may improve the magnitude and duration of anti-TB immunity, thus providing direct benefit to participants in the delayed vaccination arm [19-24].
- The incidence of vaccination-induced complications, including BCG-induced abscesses, suppurative lymphadenitis and osteomyelitis are reduced by approximately one third in infants who receive BCG vaccination after the neonatal period [21].

All infants in the study, whether in the early or delayed BCG group will benefit from regular physician reviews and free access to medical review and treatment if participants become unwell. They will also benefit from receiving all other primary vaccinations at the correct time as part of the study. The most recent survey of vaccination rates in Uganda showed that 56% of infants have not received their first set of primary immunisations (diphtheria/tetanus/pertussis/hepatitis B/*Haemophilus influenzae* (HiB) and oral polio vaccine) by 12 weeks of age, with 26% still not having received it by 1 year of age. This produces a substantial risk for those children of contracting serious, preventable illnesses, which participation in the study will negate.

Thus, we believe the general benefits of taking part in the study will outweigh the extremely small risks from a 6-week delay in BCG vaccination. The full risk-benefit analysis for this study can be found in Additional file 1.

This trial has been approved by ethics boards at the Uganda Virus Research Institute on AIDS (Ref: GC/127/13/11/432), the Uganda National Council for Science and Technology (Ref: HS 1524), The Office of the President of Uganda and the London School of Hygiene and Tropical Medicine (Ref: 6545). This study will be conducted according to the principles of the Declaration of Helsinki.

### Study limitations

The primary immunisation schedule imposes a number of constraints on the design of this study, as blood samples need to be timed to limit the potentially confounding influence of non-BCG vaccinations on innate immune responses. This is particularly relevant for comparison of the longer-term non-specific effects of BCG between the Early and Delayed intervention arms at 10 weeks, where BCG will have been given more recently in the Delayed intervention arm. As we are investigating the acute response to non-tuberculous stimulants, we believe that this should not be a problem, as any bystander effect of BCG vaccination itself is likely to be lost by 4 weeks of age. However, we are actively seeking funding for a longer-term follow-up time-point that should help to clarify this issue as well as to provide information about the duration of any non-specific effects of BCG vaccination on the innate immune system.

Although it is important to understand the biological mechanism underlying any non-specific effects of BCG vaccination, ultimately the impact on all-cause clinical illness episodes and mortality will be the outcome measures that are likely to have impacts on public healthcare policy. This study has limited power to detect differences in such outcomes, due to its small sample size. However, if suggested by the immunological and preliminary clinical data in this study, additional funding will be sought to expand the cohort to allow full interrogation of clinical outcomes.

## Discussion

Global acceptance of the hypothesis that BCG immunisation affords non-specific protective effect when given during infancy has been limited due to paucity of randomised controlled trial data and because of a lack of a confirmed biological mechanism to explain such effects in the neonatal period. We aim to address these issues by carrying out this randomised controlled trial in Uganda, providing variety of location and research group from much of the previous work, and investigating the impact of BCG immunisation on the innate immune system in neonates. Interrogation of the possible heterologous protection afforded by BCG immunisation, and the optimal timing of immunisation to achieve beneficial effects, is important to ensure that any new anti-TB vaccine or alteration in timing of BCG administration is evaluated in terms of overall benefit to recipient, rather than solely in terms of TB-specific protection alone.

### Trial status

The study commenced recruitment in September 2014. Two hundred and forty participants had been recruited as of March 2015. The trial is projected to complete recruitment by August 2015.

## Additional file

Additional file 1:
**Risk-benefit analysis of altering BCG vaccination from birth to 6 weeks of age.**

## Abbreviations

BCG: Bacillus Calmette-Guérin
*C. albicans*: *Candida albicans*
CpG: C-phosphate-G
DSMB: Data Safety Monitoring Board
DTP: Diphtheria Tetanus Pertussis
EDTA: Ethylenediaminetetraacetic acid
ELISA: Enzyme Linked Immunosorbent Assay
*E. coli*: *Escherichia coli*
GPS: Global Positioning System
HepB: Hepatitis B
HiB: *Haemophilus Influenzae B*
H3K4: Histone-3 lysine 4
ISRCTN: International Standard Randomised Controlled Trial Number
IFN-γ: Interferon gamma
IL: Interleukin
OPV: Oral Polio Vaccine
OR: Odds ratio
PBMC: Peripheral blood mononuclear cells
Poly I:C: Polyinosinic:polycytidylic acid
qPCR: Quantitative polymerase chain reaction
SAE: Serious Adverse Event
*S. aureus*: *Staphylococcus aureus*
*S. pneumonia*: *Streptococcus pneumonia*
SSI: Statens Serum Institute
SUSAR: Suspected Unexpected Serious Adverse Reactions
TB: Tuberculosis
Th1: T-helper type 1
Th2: T-helper type 2
TIBC: Total Iron Binding Capacity
TNF-α: Tumour necrosis factor-alpha
TSAT: Transferrin Saturation
TSC: Trial Steering Committee
UIBC: Unbound Iron Binding Capacity
UVRI: Uganda Virus Research Institute
