# Correction to: Investigating the non-specific effects of BCG vaccination on the innate immune system in Ugandan neonates: study protocol for a randomised controlled trial

**DOI:** 10.1186/s13063-020-04594-7

**Published:** 2020-08-10

**Authors:** Sarah Prentice, Emily L. Webb, Hazel M. Dockrell, Pontiano Kaleebu, Alison M. Elliott, Stephen Cose

**Affiliations:** 1 Wellcome Trust - Bloomsbury Centre for Global Health Research, London School of Hygiene and Tropical Medicine, Keppel Street, London, WC1E 7HT UK; 2 Clinical Research Department, London School of Hygiene and Tropical Medicine, Keppel Street, London, WC1E 7HT UK; 3 Department of Infectious Disease Epidemiology, London School of Hygiene and Tropical Medicine, Keppel Street, London, WC1E 7HT UK; 4 Department of Infection and Immunology, London School of Hygiene and Tropical Medicine, Keppel Street, London, WC1E 7HT UK; 5 MRC/Uganda Virus Research Institute on AIDS, Plot 51-59, Nakiwogo Road, PO Box 49, Entebbe, Uganda

**Correction to: Trials 16, 149 (2015)**

**https://doi.org/10.1186/s13063-015-0682-5**

Following publication of the original article [1], the authors notified us that ‘Physician-diagnosed infectious disease’ was incorrectly listed by them as a secondary outcome. In fact it is a co-primary outcome (the primary outcome for ‘Combined Studies - Clinical Illness Events’) and it is clearly listed as such in the protocol approved by the trial’s ethics committee.
