# Supplementary material for: Investigating the non-specific effects of BCG vaccination on the innate immune system in Ugandan neonates: study protocol for a randomised controlled trial
Source: Trials. 2015 Apr 11;16:149. doi: 10.1186/s13063-015-0682-5 (PMC4413988; doi:10.1186/s13063-015-0682-5)
Supplement: Additional file 1: — Risk-benefit analysis of altering BCG vaccination from birth to 6 weeks of age. [file 13063_2015_682_MOESM1_ESM.pdf]

Risk-benefit analysis of altering BCG vaccination from birth to 6 weeks of age

As BCG vaccination at birth is the standard of care in Uganda, below we detail a risk-benefit analysis for altering this standard and delaying BCG vaccination to 6 weeks of age in this population.

Benefits of delaying BCG vaccination to 6 weeks of age

- *Reduced risks of BCG induced complications*

BCG vaccination can induce a number of complications including abscesses (1/100), supurative lymphadenitis (1/1000) and osteomyelitis (1/3000) [1]. These risks are estimated to be reduced by approximately one third if vaccination is delayed past the neonatal period [2].Thus if all 560 infants in our study were vaccinated at birth we would expect approximately:

5.6 cases of BCG induced abscess formation

0.56 cases of supurative lymphadenitis

0.19 cases of BCG induced osteomyelitis

Thus if the risk of these complications is one-third less in the half of infants vaccinated at 6 weeks we would expect to save 1 case of BCG abscess formation, 0.1 cases of supurative lymphadenitis and 0.03 cases of osteomyelitis. Although small in real numbers, it should be noted that the estimated reduction in BCG induced complications in our study is higher than the estimated risk of acquiring latent or active TB (see below).

- *Potentially enhanced long-term protection against TB*

It is well documented that BCG immunisation has variable efficacy worldwide and clinical protection remains sub-optimal in most areas where neonatal BCG immunisation is recommended [3]. The recommendation for administration of BCG vaccination at birth is a historical one, based on the findings of some benefit against TB compared to un-vaccinated children and for logistical reasons as the majority of infants are reviewed by a health care professional soon after birth [4]. The optimal timing for BCG administration with respect to best long-term protection against TB has never been determined [5]. Several research groups have hypothesized that delaying BCG immunisation to a time when the immune system is more mature may produce enhanced immune responses and

therefore better clinical protection against TB [2, 6-10]. A variety of lines of immunological evidence suggest that delaying BCG immunisation to between 1 and 3 months of age results in enhanced anti-tuberculosis immune responses, including larger scar formation [2, 8] enhanced tuberculin[8]/PPD[2] reactivity *in vivo* and longer duration of PPD[9]/tuberculin[8] reactivity. One study did not find significant differences in IFN-γ secretion or proliferative response when BCG vaccination was delayed past the neonatal period [11], however it is suggested that measurement of IFN-γ underestimates the complexity of the BCG-induced Th1 response [6]. A more comprehensive study investigating other Th1 cytokines including TNF- α and IL-2, showed enhanced type 1 cytokine and memory T-cell responses when BCG vaccination was delayed to 10 weeks of age [6]. A further small study also showed a trend toward higher *in vitro* cytotoxicity and proliferative immune responses when BCG vaccination was delayed to 10 weeks [7]. It is difficult to quantify the degree of clinical TB protection that these enhanced immune responses might produce, as long-term follow-up has not been conducted in any of the above studies, but on balance they suggest that BCG vaccination efficacy may be improved if immunization is delayed. As an example, if the improved immunological outcomes resulting from delaying BCG vaccination to 6 weeks of age resulted in a reduction in the annual infection rate of infants by 10%, then 2860 infants per year would be saved from infection. (Annual infection rate = 0.02, 10% of this = 0.002 x 1430000 (live birth rate [12]) = 2860). This equates to a reduction in annual TB infection of 0.6 infants in the delayed BCG arm of our study.

Risks of delaying BCG vaccination to 6 weeks of age

- *6 weeks of potential exposure to tuberculosis without BCG vaccination*

  The following evidence suggests that the actual risk to study subjects arising from 6 weeks exposure to tuberculosis without BCG vaccination is small:

  1) Perinatal TB (TB developing from birth to 8 weeks of age) is extremely rare, with less than 300 cases worldwide described in the literature [13]. The vast majority of these cases are more appropriately described as congenital TB because the source case is the mother. In our study we will exclude infants born to mothers with known active TB or who have any positive TB screening symptoms, to further reduce this risk.
  2) A recent study using an Entebbe based birth-cohort showed a prevalence of latent- TB infection of 9.7% in children under-5 years old [14]. This gives an annual risk of infection of 1.94%, although previous studies argue that this risk is likely to be less in very young infants [15]. In our 560 study infants this equates to a potential 10.9 children/year infected (560 x 0.0194 annual risk of infection). During the 6 week period where half our study infants will not be BCG vaccinated, the likely number of infants at risk of

developing latent TB infection is thus 0.63 (10.9 children / year = 1.26 infants in 6 weeks /2 as only half infants will be unvaccinated = 0.63 children). In the above study, the strongest risk factor for latent TB infection was a known contact with a TB case (OR 2.62 (1.29-5.30), unpublished data). Thus, by our active exclusion of children with such an exposure even this small risk will be reduced (more than halved).

3) Published studies describing episodes of potential nosocomial TB transmission to neonates on neonatal intensive care units or maternity wards (from other congenitally infected infants, or from active TB in a healthcare worker), showed only 2 cases of infection out of 2603 exposed, BCG unvaccinated, infants who did not receive post-exposure prophylaxis [16-19]. The remaining infants all had negative TST and no signs of clinical disease when followed-up from 3-6 months [20]. This would suggest that if every unvaccinated infant in our study was exposed to TB, we could expect 0.22 cases of TB disease to result ((2/2603)x280). However, the infants described in these studies are likely to be much more vulnerable to infection and disease than our study subjects due to prematurity, low birth weight and existing medical conditions required their admission. The duration of exposure to the infective contact is also likely to have been much more prolonged in nosocomially infected infants that are nursed continuously in the same room as the source case, than the infants in our study would be exposed to in the community. Exclusion of infants born into families with a known case of active TB, or for whom a member of the family has any positive TB screening symptoms, will reduce the risks of similar prolonged exposures in our study. Thus the actual infection rate per exposure to TB in the delayed vaccination group is likely to be negligible.
4) At least seven previous studies have been conducted in areas of high TB prevalence that randomised infants to delayed BCG vaccination past 6 weeks of age [2, 6-10]. None of these studies showed an increase in TB incidence in the delayed vaccination group, either prior to vaccination or during follow-up (cumulative n for delayed BCG vaccination = 849, median follow-up period 1 year).

Although we believe the risks of acquiring TB due to a 6-week delay in vaccination are negligible, we have a number of measures in place to guard against any possible detriment. All infants will be closely followed up for signs and symptoms of TB infection by weekly telephone interviews and regular clinical reviews. Furthermore, any child with delayed BCG vaccination will have this flagged up on their vaccination cards so that when seen by healthcare professionals after the study they are alerted to the delay. Contact details for the study team will be included next to the alert, with a request to be notified if the subject is treated for TB after exiting the study. Although not feasible during the PhD period, we are actively seeking funds to include a longer-term follow-up point in this study, e.g. at 1 year, which could involve assessment for latent TB and treatment of any cases

found.

- *Risk of infants in the delayed arm remaining unvaccinated if lost to follow-up*

Ensuring minimal loss to follow-up is a priority in this study, particularly in the delayed BCG group. All mothers and infants will be driven home from hospital to ensure correct documentation of address and contact details. A field worker will be employed solely to contact non-attenders, by telephone and/or in person, to request clinic attendance. This will be done on a weekly basis. Transport costs for routine clinic attendance will be reimbursed (10,000 Ugandan Shillings per visit) to reduce barriers to presentation. Any BCG naïve infant who drops-out of the study will be offered BCG immunisation immediately, either at the clinic or at their home. Lastly mothers will not be blinded to vaccine administration status to avoid confusion if un-traceable subjects lost to follow-up are seen in community immunisation clinics. The BCG vaccination status will also be clearly visible in their study card and child health record.

- *If the null hypothesis is incorrect and BCG vaccination does produce a difference in the innate immune response to heterologous pathogens, then infants receiving BCG vaccination at 6 weeks of age might have a 6-week period of increased risk from all-cause infections.*

We are proposing to conduct this study because there is equipoise in the literature as to whether the non-specific effects of BCG vaccination exist, and no currently proposed biological mechanism. The main evidence suggesting a beneficial effect on all-cause mortality comes from Guinea Bissau (one randomised controlled trial in a selected population of low-birth weight infants [21] (delayed BCG n= 1161), multiple observational studies[22-28]). All other published randomised controlled trials delaying BCG vaccination past 6 weeks of age in high mortality areas have reported no difference in rates of mortality, serious illness or TB (delayed BCG subjects combined n=849). Three large cohort studies have in fact shown the reverse; that all-cause mortality is reduced when BCG is given at the same time as DTP (as it would be in the delayed arm of our study) [29]. These would suggest a benefit to all-cause mortality of *delaying* BCG administration. Thus, it is as yet unclear whether delaying BCG from birth improves, has no effect on, or worsens all-cause mortality, underlining the need for a large, well-conducted randomised controlled trial such as ours.

If the alternative hypothesis is correct, that neonatal BCG vaccination enhances the innate immune response to heterologous pathogens, this beneficial effect would not be present in the 6 weeks prior to BCG vaccination for half the study group. If this leads to clinical findings similar to those found in the Guinea-Bissau data, we would predict that this loss of beneficial effect would result in a 45%

increased all-cause mortality rate in the infants vaccinated at 6 weeks of age compared to those BCG vaccinated at birth. However, we feel it is extremely unlikely that the clinical impact of BCG vaccination at birth in Uganda would be as pronounced as in Guinea Bissau due to differences in the study populations. If BCG vaccination does enhance the innate immune response to heterologous pathogens then we would predict that infants with the highest exposure and susceptibility to infectious disease, and with the poorest access to treatment when unwell, would derive the greatest benefit from vaccination at birth. There are multiple reasons why these factors are more likely for the infants recruited to the Guinea Bissau study. Firstly, the baseline health status of infants in Guinea Bissau is significantly poorer than in Uganda with neonatal mortality rates nearly twice as high (NMR 44/1000 [12] compared to 28/1000 [12]). A combination of lower GDP, poorer living conditions and more limited access to health care are likely to contribute strongly to these differences. Secondly, the infants recruited into the Guinea Bissau study were all low birth weight/premature. This group is significantly more susceptible to serious infectious disease and death than the term, normal birth weight infants that we would recruit in this study. Lastly, the Guinea Bissau study had no enhanced clinical follow-up or improved access to health care facilities for its study participants. Information regarding mortality was collected during home visits at 3 days and 2, 6 and 12 months, with no contact in between. Unwell study children therefore utilised normal local health care facilities, which in Guinea Bissau are often of poor quality or difficult to access. In our study we will be conducting intensive and active follow-up of all participants with weekly telephone reviews to check the well-being of the child, review at home if unable to be contacted at weekly telephone review and 4 routine clinic visits before the age of 10 weeks. Transport costs will be reimbursed (10,000 Ugandan Shillings per visit) to reduce barriers to presentation. Subjects will have open access to free assessment by paediatric doctors and treatment at the research clinic throughout the study period. Lastly, the safety monitoring committee will conduct regular comparison of illness outcomes between the two groups, and the study stopped early if one group appears to be significantly more at risk of illness or death than the other. Thus, with these contingencies in place we strongly believe that we will be able to negate any increased mortality risk from invasive infectious disease that might potentially arise from altered BCG vaccination timing if the null hypothesis is incorrect.

Benefits of participation in the study for all study participants

- All participants will have regular medical follow-up with four routine clinical reviews by a paediatrician in the first 10 weeks of life. The health of study subjects is therefore likely to be improved due to early recognition and treatment of congenital conditions and serious illnesses.
- All participants will have rapid access to extra medical review and treatment if unwell
- All participants will receive primary vaccinations at the correct time. The most recent study

looking at vaccination rates in Uganda showed that 56% of infants have not received their first set of primary immunisations (diphtheria/tetanus/pertussis/hepatitis B/Haemophilus influenza/polio) by 12 weeks of age, with 26% still not having received it by 1 year of age. This produces a substantial risk for those children of contracting these preventable illnesses that are extremely dangerous in infants. Thus, by ensuring all subjects in our study receive primary immunisations at the correct time we would estimate that we would provide considerable benefits by reducing the risk of these infectious diseases in approximately 314 infants. These benefits alone are likely to outweigh the extremely low increased risk of TB infection in the delayed vaccination group.

- All costs of transport to attend the clinic and receive vaccinations will be reimbursed. This is likely to improve healthcare utilisation.
- All mothers will receive education about the importance of vaccinations and other basic infant and child health messages.

Potential benefits of the research for child health in Uganda and globally

- If BCG vaccination does provide heterologous protection against non-mycobacterial invasive infectious disease, ensuring that every child is immunised with BCG vaccination as soon as possible after birth could save many lives. In Uganda, for example, although it is recommended that BCG should be received at birth, a recent survey showed that approximately 50% of infants had not received it by 12 weeks of age [30]. The only currently published estimate of the heterologous protection provided by BCG vaccination at birth compared to delayed is a 45% reduction in mortality rate in the first 4 weeks of life (Guinea-Bissau data) [21]. Thus, if we assume that infants in Uganda would have the same mortality reduction, then ensuring that the 50% of infants not currently vaccinated by 12 weeks are vaccinated at birth would prevent 22,800 neonatal deaths per year in Uganda alone. (Current neonatal mortality rate in Uganda = 28/1000. If a 45% higher mortality rate in unvaccinated neonates is assumed then the differential neonatal mortality rates would be 36/1000 for unvaccinated infants and 20/1000 for vaccinated infants. Thus, ensuring all unvaccinated infants are vaccinated would save 16/1000 deaths per year. With an annual birth rate of 1.43 million, this gives a saving of 22880 neonatal deaths per year). A study comparing 45 low and middle income countries showed that on average 50% of children will not have received BCG vaccination by 4 weeks of age. BCG vaccination is also being phased out in many countries with low TB incidence. As the average global neonatal mortality rate is 36/1000, applying the same logic as before and assuming 50% of children globally will not have received BCG vaccination by 4 weeks of age, this would give a differential global neonatal mortality rate of 26/1000 for BCG vaccinated and 46/1000 for BCG unvaccinated infants. Thus, ensuring all 141 million infants born globally each year are vaccinated at birth could potentially save 2.82 million deaths per year.

- This study aims to improve our knowledge of the developing infant immune system up to 10 weeks of age. The current understanding of the developing infant immune system is limited. Increasing our understanding may have broad reaching implications for global child health which are currently difficult to quantify.

- This study aims to improve our understanding of the potential non-specific effects of vaccinations. If our hypothesis proves correct it would suggest the need for all vaccinations to be evaluated on the basis of impacts on all-cause mortality/morbidity rather than just disease-specific outcomes. Again this has the potential to have substantial, and as yet unquantifiable, impacts on the global health of children.

Thus, in summary, we believe that delaying BCG vaccination in this study poses negligible risks of increased TB infection. Although if the null-hypothesis is not correct it could pose a risk from non-TB infectious disease in the delayed vaccination group, we believe this risk would be slight and negated by our active and intensive clinical follow-up. If BCG vaccination at birth does protect against heterologous invasive infectious disease it would have profound impacts on global child health, which we believe outweigh these small risks.

Evaluation of alternative study designs

During the development of this proposal, other study designs were considered. However, there are several reasons why we have concluded that a randomized controlled trial design, as described, is imperative for this work.

a) *Observational studies, particularly involving vaccinations, are highly susceptible to selection bias.*

It is argued in the literature that children who have delayed vaccinations are likely to be fundamentally different to those that receive their vaccinations at the recommended times, confounding the interpretation of the results of this study [31]. Such differences include:

- Lower socioeconomic status [32]
- Lower educational status of parents [32]
- Poorer nutritional status [23]
- Parents less pro-active about the health of their infants, or less able to access healthcare facilities [32]
- More likely to be born at home with unskilled birth attendants rather than in hospital [33]

It is also recognized that observational studies comparing children vaccinated at the recommended time with those where vaccination has been delayed are subject to frailty bias, as vaccinations tend to be delayed in acutely unwell or chronically sick children and those of low birth weight [31].

All of the above factors are likely to impact on the immunological status of the child and increase their risk of clinical illness episodes, due to factors such as increased exposure to infectious pathogens from less hygienic living conditions and overcrowding, as well as altered healthcare seeking behavior/access negatively influencing illness episode outcomes.

Thus, the likely predominance of healthier neonates in the vaccinated group may bias both the primary immunological outcome measures and our clinical illness data toward making BCG vaccination at birth appear more beneficial against all-cause morbidity and mortality than it really is.

Although it is possible to document and adjust for the recognized variables associated with delayed vaccine receipt, it is strongly argued in the literature that it is impossible to anticipate all confounding variables separating the two groups. Multiple observational studies have been conducted, including case-control studies and prospective/retrospective cohort analyses, investigating the potential non-

specific effects of a range of vaccinations (reviewed in [34]) These have had minimal acceptance by the research community and public health policy makers for the above reasons. In a review of the potential non-specific effects of vaccinations, the Global Advisory Committee on Vaccines has stated that ‘conclusive evidence for or against non-specific effects of vaccines on mortality is unlikely to be obtained from observational studies’ [35].

Thus, we believe that the information gained from an observational study would be insufficient to accept or refute our hypothesis, would not significantly add to what is already known in this field, and would have little impact on public healthcare policy in Uganda and worldwide. We therefore argue that it may be less ethically justifiable to subject infants to blood draws and commit research funds and resources to an observational study, when we believe a randomized controlled trial delaying BCG vaccination may be safely carried out [4].

b) Inability to collect baseline cord blood, early neonatal blood samples and early clinical outcome data if we passively recruit infants attending vaccination clinics who have missed vaccination at birth.

The evidence from Guinea-Bissau suggests that the major benefit from BCG vaccination at birth is derived within the first week of life [36]. It would be impossible in an observational study that recruits non-BCG vaccinated infants that are attending community vaccination clinics (with attendance normally at 6 weeks of age for their first primary immunisations) to compare immunological and clinical data between the two groups at these vital early time-points.

References

1. Institute SS: **BCG-Danish Product Characteristic Leaflet.** 2013.
2. Ildirim I, Sapan N, Cavusoglu B: **Comparison of BCG vaccination at birth and at third month of life.** *Archives of disease in childhood* 1992, **67:**80-82.
3. Price JF: **BCG vaccination.** *Archives of disease in childhood* 1982, **57:**485-486.
4. Shann F, Nohynek H, Scott JA, Hesseling A, Flanagan KL, Working Group on Nonspecific Effects of V: **Randomized trials to study the nonspecific effects of vaccines in children in low-income countries.** *The Pediatric infectious disease journal* 2010, **29:**457-461.
5. **BCG vaccination policies. Report of a WHO study group.** *World Health Organization technical report series* 1980, **652:**1-17.
6. Kagina BM, Abel B, Bowmaker M, Scriba TJ, Gelderbloem S, Smit E, Erasmus M, Nene N, Walzl G, Black G, et al: **Delaying BCG vaccination from birth to 10 weeks of age may result in an enhanced memory CD4 T cell response.** *Vaccine* 2009, **27:**5488-5495.
7. Hussey GD, Watkins ML, Goddard EA, Gottschalk S, Hughes EJ, Iloni K, Kibel MA, Ress SR: **Neonatal mycobacterial specific cytotoxic T-lymphocyte and cytokine profiles in response to distinct BCG vaccination strategies.** *Immunology* 2002, **105:**314-324.
8. Suciliene E, Ronne T, Plesner AM, Semenaite B, Slapkauskaite D, Larsen SO, Haslov K: **Infant BCG vaccination study in Lithuania.** *The international journal of tuberculosis and lung disease : the official journal of the International Union against Tuberculosis and Lung Disease* 1999, **3:**956-961.
9. Pabst HF, Godel JC, Spady DW, McKechnie J, Grace M: **Prospective trial of timing of bacillus Calmette-Guerin vaccination in Canadian Cree infants.** *The American review of respiratory disease* 1989, **140:**1007-1011.
10. Ota MO, Vekemans J, Schlegel-Haueter SE, Fielding K, Sanneh M, Kidd M, Newport MJ, Aaby P, Whittle H, Lambert PH, et al: **Influence of Mycobacterium bovis bacillus Calmette-Guerin on antibody and cytokine responses to human neonatal vaccination.** *Journal of immunology* 2002, **168:**919-925.
11. Burl S, Adetifa UJ, Cox M, Touray E, Ota MO, Marchant A, Whittle H, McShane H, Rowland-Jones SL, Flanagan KL: **Delaying bacillus Calmette-Guerin vaccination from birth to 4 1/2 months of age reduces postvaccination Th1 and IL-17 responses but leads to comparable mycobacterial responses at 9 months of age.** *Journal of immunology* 2010, **185:**2620-2628.
12. **Central Intelligence Agency - World Factbook**
13. Whittaker E, Kampmann B: **Perinatal tuberculosis: new challenges in the diagnosis and treatment of tuberculosis in infants and the newborn.** *Early human development* 2008, **84:**795-799.
14. Nkurunungi G, Lutangira JE, Lule SA, Akurut H, Kizindo R, Fitchett JR, Kizito D, Sebina I, Muhangi L, Webb EL, et al: **Determining Mycobacterium tuberculosis infection among BCG-immunised Ugandan children by T-SPOT.TB and tuberculin skin testing.** *PloS one* 2012, **7:**e47340.
15. Stott H, Patel A, Sutherland I, Thorup I, Smith PG, Kent PW, Rykushin YP: **The risk of tuberculous infection in Uganda, deprived from the findings of national tuberculin surveys 1958 and 1970.** *Tubercle* 1973, **54:**1-22.
16. Light IJ, Saidleman M, Sutherland JM: **Management of newborns after nursery exposure to tuberculosis.** *The American review of respiratory disease* 1974, **109:**415-419.

17. Steiner P, Rao M, Victoria MS, Rudolph N, Buynoski G: **Miliary tuberculosis in two infants after nursery exposure: epidemiologic, clinical, and laboratory findings.** *The American review of respiratory disease* 1976, **113:**267-271.
18. Burk JR, Bahar D, Wolf FS, Greene J, Bailey WC: **Nursery exposure of 528 newborns to a nurse with pulmonary tuberculosis.** *Southern medical journal* 1978, **71:**7-10.
19. Myers JP, Perlstein PH, Light IJ, Towbin RB, Dincsoy HP, Dincsoy MY: **Tuberculosis in pregnancy with fatal congenital infection.** *Pediatrics* 1981, **67:**89-94.
20. Laartz BW, Narvarte HJ, Holt D, Larkin JA, Pomputius WF, 3rd: **Congenital tuberculosis and management of exposures in a neonatal intensive care unit.** *Infection control and hospital epidemiology : the official journal of the Society of Hospital Epidemiologists of America* 2002, **23:**573-579.
21. Aaby P, Benn CS: **Non-specific and sex-differential effects of routine vaccines: what evidence is needed to take these effects into consideration in low-income countries?** *Human vaccines* 2011, **7:**120-124.
22. Garly ML, Martins CL, Bale C, Balde MA, Hedegaard KL, Gustafson P, Lisse IM, Whittle HC, Aaby P: **BCG scar and positive tuberculin reaction associated with reduced child mortality in West Africa. A non-specific beneficial effect of BCG?** *Vaccine* 2003, **21:**2782-2790.
23. Kristensen I, Aaby P, Jensen H: **Routine vaccinations and child survival: follow up study in Guinea-Bissau, West Africa.** *Bmj* 2000, **321:**1435-1438.
24. Roth A, Garly ML, Jensen H, Nielsen J, Aaby P: **Bacillus Calmette-Guerin vaccination and infant mortality.** *Expert review of vaccines* 2006, **5:**277-293.
25. Roth A, Sodemann M, Jensen H, Poulsen A, Gustafson P, Weise C, Gomes J, Djana Q, Jakobsen M, Garly ML, et al: **Tuberculin reaction, BCG scar, and lower female mortality.** *Epidemiology* 2006, **17:**562-568.
26. Rodrigues A, Fischer TK, Valentiner-Branth P, Nielsen J, Steinsland H, Perch M, Garly ML, Molbak K, Aaby P: **Community cohort study of rotavirus and other enteropathogens: are routine vaccinations associated with sex-differential incidence rates?** *Vaccine* 2006, **24:**4737-4746.
27. Stensballe LG, Nante E, Jensen IP, Kofoed PE, Poulsen A, Jensen H, Newport M, Marchant A, Aaby P: **Acute lower respiratory tract infections and respiratory syncytial virus in infants in Guinea-Bissau: a beneficial effect of BCG vaccination for girls community based case-control study.** *Vaccine* 2005, **23:**1251-1257.
28. Veirum JE, Sodemann M, Biai S, Jakobsen M, Garly ML, Hedegaard K, Jensen H, Aaby P: **Routine vaccinations associated with divergent effects on female and male mortality at the paediatric ward in Bissau, Guinea-Bissau.** *Vaccine* 2005, **23:**1197-1204.
29. Higgins JPT S-WK, Reingold A: **Systematic review of the non-specific effects of BCG, DTP and measles containing vaccines.** *WHO SAGE Review*
2014.
30. Clark A, Sanderson C: **Timing of children's vaccinations in 45 low-income and middle-income countries: an analysis of survey data.** *Lancet* 2009, **373:**1543-1549.
31. Fine PE, Williams TN, Aaby P, Kallander K, Moulton LH, Flanagan KL, Smith PG, Benn CS, Working Group on Non-specific Effects of V: **Epidemiological studies of the 'non-specific effects' of vaccines: I--data collection in observational studies.** *Tropical medicine & international health : TM & IH* 2009, **14:**969-976.

32. Jahn A, Floyd S, Mwinuka V, Mwafilaso J, Mwagomba D, Mkisi RE, Katsulukuta A, Khunga A, Crampin AC, Branson K, et al: **Ascertainment of childhood vaccination histories in northern Malawi.** *Tropical medicine & international health : TM & IH* 2008, **13:**129-138.
33. Cutts FT, Rodrigues LC, Colombo S, Bennett S: **Evaluation of factors influencing vaccine uptake in Mozambique.** *International journal of epidemiology* 1989, **18:**427-433.
34. Shann F: **The nonspecific effects of vaccines and the expanded program on immunization.** *The Journal of infectious diseases* 2011, **204:**182-184.
35. **Meeting of Global Advisory Committee on Vaccine Safety, 18-19 June 2008.** *Releve epidemiologique hebdomadaire / Section d'hygiene du Secretariat de la Societe des Nations = Weekly epidemiological record / Health Section of the Secretariat of the League of Nations* 2008, **83:**287-292.
36. Aaby P, Roth A, Ravn H, Napirna BM, Rodrigues A, Lisse IM, Stensballe L, Diness BR, Lausch KR, Lund N, et al: **Randomized trial of BCG vaccination at birth to low-birth-weight children: beneficial nonspecific effects in the neonatal period?** *The Journal of infectious diseases* 2011, **204:**245-252.
